# Skeletal muscle-derived cell implantation for the treatment of sphincter-related faecal incontinence

**DOI:** 10.1186/s13287-018-0978-y

**Published:** 2018-09-13

**Authors:** Andrea Frudinger, Rainer Marksteiner, Johann Pfeifer, Eva Margreiter, Johannes Paede, Marco Thurner

**Affiliations:** 1 Department of Obstetrics and Gynaecology, Division of Gynaecology, Medical University of Graz, Auenbruggerplatz 14, 8036 Graz, Austria; 2 Innovacell Biotechnologie AG, Science Park, Innsbruck, Austria; 3 Department of General Surgery, Medical University of Graz, Graz, Austria; 4 B-K Ultrasound, Pascalkehre 13, 25451 Quickborn, Germany

**Keywords:** Faecal incontinence, Anal sphincter, Cell therapy, Autologous skeletal muscle derived cells

## Abstract

**Background:**

In an earlier pilot study with 10 women, we investigated a new approach for therapy of faecal incontinence (FI) due to obstetric trauma, involving ultrasound-guided injection of autologous skeletal muscle-derived cells (SMDC) into the external anal sphincter (EAS), and observed significant improvement. In the current study, we tested this therapeutic approach in an extended patient group: male and female patients suffering from FI due to EAS damage and/or atrophy. Furthermore, feasibility of lower cell counts and cryo-preserved SMDC was assessed.

**Methods:**

In this single-centre, explorative, baseline-controlled clinical trial, each patient (*n* = 39; mean age 60.6 ± 13.81 years) received 79.4 ± 22.5 × 10^6^ cryo-preserved autologous SMDC. Changes in FI parameters, Fecal Incontinence Quality of Life (FIQL), anorectal manometry and safety from baseline to 1, 6 and 12 months post implantation were evaluated.

**Results:**

SMDC used in this trial contained a high percentage of myogenic-expressing (CD56^+^) and muscle stem cell marker-expressing (Pax7^+^, Myf5^+^) cells. Intervention was well tolerated without any serious adverse events. After 12 months, the number of weekly incontinence episodes (WIE, primary variable), FIQL and patient condition had improved significantly. In 80.6% of males and 78.4% of females, the WIE frequency decreased by at least 50%; Wexner scores and severity of FI complaints decreased significantly, independent of gender and cause of FI.

**Conclusions:**

Injection of SMDCs into the EAS effectively improved sphincter-related FI due to EAS damage and/or atrophy in males and females. When confirmed in a larger, placebo-controlled trial, this minimal invasive procedure has the potential to become first-line therapy for FI.

**Trial registration:**

EU Clinical Trials Register, EudraCT 2010-023826-19 (Date of registration: 08.11.2010).

## Background

Therapeutic options for faecal incontinence (FI) are manifold [1, 2]. Conservative measures such as biofeedback techniques or dietary changes can be offered to individuals suffering from FI. They might be ineffective so that ultimately surgical interventions are needed. The most common surgical procedure is overlapping sphincter repair to restore mechanical integrity. The outcome is suboptimal and deteriorates most of the time within only 5 years. Sacral nerve modulation is now favoured but requires high patient compliance as pacemakers have to be readjusted and changed from time to time. Even today a permanent stoma often remains a final solution. Non-surgical procedures such as the injection of bulking agents remain to be evaluated as their results are very variable as well as their complications and side effects. Thus, the need for alternative approaches to meet therapeutic aims is high [3–11].

A new approach that might meet this need is cell therapy using autologous cells to restore functionality of muscular structures [12]. This approach is based on the isolation of quiescent satellite cells from skeletal muscle biopsies that can be activated to re-enter the cell cycle (muscle stem cells), and by asymmetric division give rise to new satellite cells as well as symmetrically dividing myogenic progenitor cells. Myogenic progenitor cells either proliferate or become terminally differentiated myocytes, able to fuse with each other and/or existing myofibres [13]. Implantation of these skeletal muscle-derived cells (SMDC) has been used in several therapeutic settings, including myocardial infarction [14], Duchenne muscular dystrophy [15] and stress urinary incontinence [16], with mixed results. However, only few studies and preliminary results for the treatment of FI are available [17–20]. In an earlier pilot study in 10 women [17, 18], we evaluated the technical feasibility and safety of ultrasound-guided injection of autologous SMDC into the external anal sphincter (EAS) to treat FI due to obstetric trauma. Injection was very well tolerated and led to highly significant reductions in Wexner score and bowel movements as well as a marked improvement in the Fecal Incontinence Quality of Life (FIQL) score [21] after 1 year. A follow-up after 5 years demonstrated a sustained improvement of FI episodes, physiological measurements of anal function and FIQL [18]. Another study of 10 patients (one male, nine female) with FI due to sphincter insufficiency of various origins reported improvements (Wexner score, Fecal Incontinence Severity Index (FISI)) in four patients after 1 year following injection of autologous muscle-derived stem cells [20]. Just recently, a small placebo-controlled phase II study on 24 women who had also received autologous SMDC or placebo for therapy of FI was published [19]. The outcome was quite positive: 1 year post implantation, the Cleveland Clinical Incontinence (CCI) score had significantly decreased compared to placebo and the quality of life (QOL) improved significantly, at least regarding selected parameters of the FIQL score (lifestyle, coping/behaviour). All of these studies clearly indicate SMDC implantation to be a promising approach for treatment of FI.

The previous studies mostly included patients suffering from FI due to EAS damage, such as obstetric trauma, or patients with highly heterogeneous causes of FI [17–20]. However, a large proportion of patients suffer from FI because the anal sphincter becomes atrophic [22, 23]. Often caused by degenerative processes, this condition is particularly common in older people [24–26], but FI might also occur earlier due to atrophy and preceding EAS damage. Thus, in the current study, we wanted to test the efficacy of ultrasound-guided SMDC implantation into the EAS in an extended target group: patients suffering from FI associated with EAS damage and/or EAS atrophy.

In our pilot study [17] and another publication [20] on cell injection for FI therapy, patients received cells directly after harvest and simple chilling on ice. From the third study on cell implantation for FI therapy, however, very preliminary promising data from a small subgroup (*n* = 10) on injection of cryo-preserved cells are available [19]. Thus, another aim of the current study was to investigate whether cryo-preserved autologous SMDC are effective in our treatment set-up. Further, we wanted to know whether a reduced number of cells (approximately 80 ± 30 × 10^6^ cells per patient) is also effective compared to previously used numbers (121 ± 12 × 10^6^ cells [17], 249 ± 68 × 10^6^ cells [20] and 100 ± 20 × 10^6^ cells [19]). Moreover, we aimed for a larger study population of about 40 patients as previous studies included a maximum of 12 patients within the ITT set [17–20]. Finally, since men and women are equally affected by FI [25, 27] and to date only one male patient has been enrolled in a similar trial [20], we also addressed male patients within our study.

## Methods

### Study design

This was a single-centre, explorative, baseline-controlled clinical trial conducted at the Department of Obstetrics and Gynecology, Medical University of Graz, Austria (start February 2011). The study is registered with the EU Clinical Trials Register (EudraCT 2010-023826-19) and was approved by the Independent Ethics Committee of the Medical University of Graz, Austria. All procedures were carried out in conformity with the principles of the Declaration of Helsinki and of the International Conference on Harmonization—Good Clinical Practice (ICH-GCP) as well as according to appropriate regulations of the local laws. Prior to performance of any protocol-specific procedures, written informed consent was obtained from eligible patients.

**Fig. 1:**
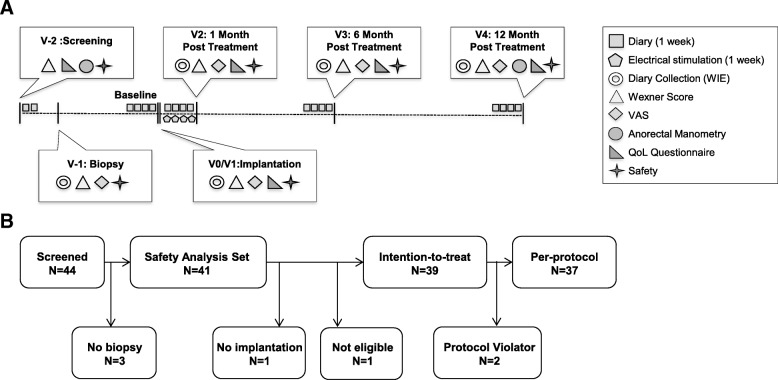
Study overview. **a** Study visits, including parameters evaluated, and timeline for data collection. **b** Number of patients screened, included in safety analysis, intention-to-treat and per-protocol groups. QoL quality of life, V visit, VAS visual analogue scale, WIE weekly incontinence episodes

For each patient, seven visits were scheduled: two visits before myoblast implantation (V-2, screening; V-1, biopsy), one for myoblast implantation (V0, implantation) and four after implantation (V1–V4, 1 day and 1, 6 and 12 months post implantation); the total period per patient was 15 months. Study visits, including evaluated parameters, and the timeline for data collection are outlined in Fig. 1.

### Study population

Participants were recruited prospectively by running an advertisement in local newspapers in Graz, Austria, stating that females and males of at least 18 years of age who suffer from FI are sought for a clinical study. The planned size of the study group was 40 patients. Major inclusion criteria were ≥ 18 years of age, FI (more than 4–5 incontinence episodes per week) for more than 6 months, as confirmed at screening by relevant medical history and anorectal examination, and Wexner incontinence score > 9. The latter was confirmed by incontinence diaries (for details, see “Incontinence diary”) kept by participants for 2 weeks before definitive recruitment at V-1. Women of childbearing age had to agree not to become pregnant during the observational period. Patients who, according to the clinical judgment of the investigator, were not suitable for inclusion due to acute anal sphincter injury including obstetric and other trauma, acute disc prolapse or neurological diseases were also excluded.

### Diagnostic procedures

Anal endosonography was performed in all patients by a single female operator (AF), who was experienced in the technique, having performed at least 2000 procedures previously. A B-K Flexfocus Type 400 Medical Scanner fitted with a 2052 endoprobe was used (B-K Medical, Herlev, Denmark). Patients were examined in the supine position using a standard technique [28]. A 3D dataset that encompassed the entire anal canal length was obtained [29].

For anorectal manometry, a ManoScan™360 High-Resolution Manometry (Sierra Scientific Instruments, Los Angeles, CA, USA) connected with a MCC-3885-3D Mano Scan anorectal catheter with 16 circumferential sensing regions and 256 total sensors, central balloon inflation lumen and 10-mm probe diameter at solid state was used.

Pudendal nerve terminal motor latency (PNTML) was measured with a Keypoint electromyography device (Dantec, Scovlunde, Denmark) and a St. Mark’s pudendal nerve electrode (Medtronic, Shoreview, MN, USA) [30]. Neuropathy was defined by latencies > 2.3 ms.

### Incontinence diary

In an incontinence diary, patients had to record the following parameters: date and time of each bowel movement; daily episodes of incontinence to stool; and urgency. Also, patients had to mark the severity of complaints caused by FI on a visual analogue scale (VAS) [31] and describe the amount of stool lost (“traces”, “a little”, “more”). Periods when patients had to keep the incontinence diary are indicated in Fig. 1a.

### Muscle biopsy and SMDC culture

A skeletal muscle biopsy to obtain autologous SMDC was taken as described previously [17]. Briefly, approximately 1 cm^3^ of skeletal muscle (*Musculus pectoralis major*) was removed and transported to a cGMP facility (Innovacell Biotechnology AG, Austria). Pure skeletal muscle tissue was enzymatically digested by incubation with collagenase (Serva/Nordmark, Germany). The SMDC obtained were maintained by standard cell culture methods. Cells were cultured in Ham’s F-10 basal medium supplemented with foetal calf serum (Life Technologies, UK) and bFGF (CellGenix, Freiburg, Germany) at 37 °C, 5% CO_2_. The medium was changed every 3–4 days. SMDC were subcultivated three times corresponding to a mean ± SD cultivation time of 29.31 ± 5 days followed by harvest, characterization and cryo-preservation. A total of 79.4 ± 22.5 million cells per patient resuspended in Ringer lactate (Fresenius Kabi, Austria) supplemented with DMSO (OriGen, TX, USA) and human serum albumin (Baxter, IL, USA) were cryo-preserved in liquid nitrogen until implantation. Residual cells not used for implantation were subsequently analysed by microarray, flow cytometry and immunocytochemistry.

### Flow cytometry

Flow cytometry analysis was performed on a Guava easyCyte 6HT 2 L flow cytometer (Merck Millpore, Darmstadt, Germany). Briefly, cells were harvested by trypsin at 37 °C for 5 min, centrifuged at 400 × *g* and resuspended in 1× PBS supplemented with 1% FCS. For surface marker staining, cells in a concentration of 40,000 cells/reaction were incubated with anti-SSEA1-PE (BD Biosciences, Pharmingen™, San Diego, CA, USA), anti-SSEA3-PE (Merck Millipore), anti-SSEA4-PE (BD Biosciences, Pharmingen™), IgG1-PE (Beckman Coulter Inc., France), anti-CD56-PE (Beckman Coulter Inc.), anti-TRA-1-60-FITC (BD Biosciences, Pharmingen™), anti-TRA-1-81-FITC (Merck Millipore), IgG1-FITC (Beckman Coulter Inc.) or anti-CD90-PE (Beckman Coulter, Inc.) for 15 min in a 1.5-ml Eppendorf tube at 4 °C in the dark. After incubation, cells were washed with 1 ml of 1× PBS, centrifuged at 400 × *g*, resuspended in 200 μl of 1× PBS and each reaction received 5 μl of viability dye 7-aminoactinomycin D (Beckman Coulter Inc.). For intracellular marker staining, 50,000 cells/reaction were centrifuged at 400 × *g* followed by resuspension in BD Cytofix/Cytoperm Fixation and Permeabilisation Solution (BD Biosciences, Pharmingen™) and incubation at 4 °C for 20 min. Afterwards, cells were washed with BD Perm/Wash Buffer (diluted 1:10 in aqua dest) (BD Biosciences, Pharmingen™) and centrifuged. Cells were then resuspended in 1× PBS and incubated with IgG1-FITC (Beckman Coulter Inc.), IgG1-PE (Beckman Coulter Inc.), anti-Oct3/4-PE (BD Biosciences, Pharmingen™), anti-Nanog-Alexa488 (BD Biosciences, Pharmingen™), anti-Sox-2-PE (BD Biosciences, Pharmingen™), anti-UTF-1-FITC (Merck Millipore) or anti-Pax-7-Alexa488 (Bioss Antibodies Inc., MA, USA) for 1 h at 4 °C in the dark. Subsequently, the cells were centrifuged, washed with BD Perm/Wash buffer (diluted 1:10 in aqua dest) and after a final centrifugation step resuspended in 1× PBS. Cell events were acquired by employing Guava InCyte™ v.2.3 software. Histograms were generated with a minimum of 3000 events with a sample flow rate of 1.8 μl/ml. The percentage of positive cells was obtained by comparison with isotype control set as 99% negative.

### Gene expression analysis

CD56^–^ non-myogenic SMDC, previously characterized as mesenchymal progenitors [32, 33], were isolated as described previously [32] and used to compare gene expression to the CD56^+^ SMDC used during the clinical trial. Total RNA of 1 million cells per sample was isolated by RNEasy Kit (QIAGEN, Hilden, Germany) according to the manufacturer’s instructions. Sample preparation for microarray hybridization was carried out as described in the NuGEN Ovation PicoSL WTA System V2 and NUGEN Encore Biotin Module manuals (NuGEN Technologies, Inc., San Carlos, CA, USA). Hybridized arrays (Human Gene 2.0 ST) were washed and stained in an Affymetrix Fluidics Station FS450, and the fluorescent signals were measured with an Affymetrix GeneChip Scanner 3000 7G. Fluidics and scan functions were controlled by Affymetrix GeneChip Command Console v4.1.3 software. Sample processing was performed at an Affymetrix Service Provider and Core Facility, “KFB—Center of Excellence for Fluorescent Bioanalytics” (Regensburg, Germany). Summarized probe set signals in log_2_ scale were calculated using the RMA algorithm with Affymetrix GeneChip Expression Console v1.4. Probe set signals of IDs annotated to genes were analysed and heat maps were generated employing Multiple Expression Viewer (MeV 3.1.0). Significance analysis of differentially expressed genes between CD56^+^ and CD56^–^ cells was performed by employing two-class unpaired significance analysis of microarrays (SAM) in MeV 3.1.0 software [34, 35]. The δ value was set to 1.0 to exclude any falsely significant genes and the log_2_ fold-change threshold was set to 1.4.

### Immunocytochemistry

SMDC seeded on 24-well plates were washed by aspirating medium and adding 1× PBS. After aspiration of PBS, 500 μl of − 20 °C pre-cooled MetOH was used to cover cells and incubated at room temperature (RT) for 10 min for fixation. After washing the cells three times with 1× PBS, cells were covered with Ultravision Hydrogen Peroxide Block (Thermo Fisher Scientific, MA, USA) and incubated for 5 min at room temperature. After three additional washing steps, cells were covered with 1:100 diluted anti-Myf5 (Santa Cruz biotechnology, TX, USA) or 1:100 diluted anti-desmin antibodies (Thermo Fisher Scientific) and incubated at 37 °C for 90 min. Cells were washed again with PBS and covered with ready-to-use biotinylated goat anti-rabbit or goat anti-mouse secondary antibodies (Thermo Fisher Scientific) and incubated for 60 min at 37 °C. Afterwards, cells were washed with 1× PBS, covered with 1:100 diluted horseradish peroxidase-conjugated streptavidin (Vector Laboratories, Inc., CA, USA) and incubated for 30 min at 37 °C. Subsequently, the cells were washed and incubated with the Lab Vision™ Ready-To-Use AEC Substrate System (Thermo Fisher Scientific) for 10 min at room temperature. The reaction was stopped by washing with PBS three times and cells were visualized on a Nikon Eclipse TE2000-U inverted Microscope. The percentage of Myf5-positive nuclei per total nuclei was calculated from micrographs of five individual experiments.

### Patient preparation and ultrasound-guided myoblast implantation

SMDC implantation was performed as described previously [17] by the principal investigator (AF). The patient was placed in a supine position and kept under general anaesthesia to avoid movement during injection. SMDC were injected under direct ultrasound guidance using the Type 400 Flexfocus scanner and Type 8848 10 MHz transducer with a specifically designed injection device (B-K Medical). The transducer allowed simultaneous biplane scanning so that implantation could be visualized in a 65 mm + 30° trapezoid sagittal plane while simultaneously viewing needle ingress in a 180° transverse plane. The tip of the transducer was lubricated with sterile ultrasound gel, covered with a sterile condom, lubricated again and inserted into the anal canal. The entire anal canal length, starting at the puborectalis sling, was scanned before injection, which was essential for the calibration of the injection device. For each patient, three vials with 1 ml of frozen cells (approximately 2.5 × 10^7^ cells/ml) were diluted with 1 ml of Ringer’s lactate each. The resulting total volume of 6 ml was administered in 12 depots (12 × 0.5 ml). Individual injections of 0.5 ml were extended in a circular array directly into the EAS. Care was taken not to inject into the longitudinal muscle, internal anal sphincter or subepithelium. For the procedure, all patients were hospitalized for 1 day.

### Concomitant therapy

Since pelvic floor electrical stimulation was shown to enhance integration of SMDC into host tissue [36, 37], all patients had to perform anal canal electrical stimulation using a Syntic Electrical Stimulation System with an anal plug (tic Medizintechnik GmbH, Germany) for 4 weeks after cell implantation starting at V1 (Fig. 1a). To guarantee adequate use of the apparatus, patients were instructed by authorized and trained hospital staff. The procedure was recorded by the apparatus, facilitating monitoring of compliance.

### Outcome measures

The aim of this trial was to analyse the efficacy and safety of an ultrasound-guided injection of expanded SMDC into the EAS for treatment of FI. The primary variable was the frequency of incontinence episodes (IE) calculated as the number of weekly incontinence episodes (WIE) over the following periods: 2 weeks before the biopsy at V-1 (day − 84 to day − 70), 4 weeks before the implantation at V0 (day − 28 to day 0), 4 weeks between the implantation and V2 (day 1 to day 28, 4 weeks after implantation), and then 4 weeks before V3 (day 140 to day 168, 6 months after implantation) and 4 weeks before V4 (day 337 to day 365, 12 months after implantation). The calculations were based on the patient’s record in the incontinence diary.

Secondary variables for efficacy were change in Wexner score, change in VAS, severity of incontinence, response to treatment, changes in anorectal manometry data, patient assessment based on FIQL score (domains: lifestyle, coping/behaviour, depression/perception, embarrassment) [21] and investigator’s assessment by Clinical Global Impression (CGI) score [38]. Severity of incontinence was evaluated by giving records in the incontinence diary on the amount of stool lost as numeric factors (1 = traces, 2 = a little, 3 = more) that were eventually summarized. Responders were defined as patients with a decrease in WIE of 100%, 50–99%, 20–49% and 0–19% as R_(100),_ R_(50–99)_, R_(20–49)_ and R_(0–19)_, respectively, throughout all study visits.

Secondary variables for safety involved physical examination (including abdomen–anal examination), vital signs, standard hematology, blood chemistry, urinalysis, pregnancy test, concomitant medication, adverse events (AEs) and serious adverse events (SAEs).

### Statistical analysis

This was an exploratory trial and no formal statistical hypothesis was investigated. The statistical analyses were performed at Pierrel Research Europe GmbH, using SAS® version 9.2 or later (SAS Institute Inc., Cary, NC, USA) on a Microsoft® Windows® 2000 Professional or subsequent platform. The primary endpoint was the number of WIE as derived from the incontinence diary. The analysis of the primary endpoint was based on the intention to treat (ITT) population. Summary statistics for the number of WIE was provided for the pre–post differences between each post-baseline visit (V2, V3 and V4) and V0. In addition, the 95% confidence interval (CI) was calculated for the mean of the pre–post differences and used to address post-hoc significance of the primary and secondary variable changes from baseline to every post-baseline visit, thereby considering a 95% CI range not including 0 as a significant change. All secondary endpoints were analysed in an exploratory way using the 95% CI to address significance in pre–post treatment changes, as for primary endpoint analysis. Descriptive summary statistics are provided for continuous variables and the respective changes (VAS, Wexner score, severity, FIQL, CGI, anorectal function parameters). For responder analysis, frequencies and percentages were provided. Safety parameters were evaluated descriptively.

## Results

### Patients’ disposition and baseline characteristics

The overall trial duration was from February 2011 until October 2012. One centre in Austria (Department of Obstetrics and Gynecology, Medical University of Graz) enrolled a total of 44 patients (Fig. 1b). Three patients did not undergo biopsy and were therefore excluded from the safety set. Two patients of the safety set (*N* = 41) were not included in the ITT population due to either receiving no implantation or being not eligible due to insufficient FI severity at screening and baseline. Two patients of the ITT set (*N* = 39) showed major protocol deviations and were excluded from the per-protocol (PP) population. Evaluation was performed for the safety set (*N* = 41), the ITT set (*N* = 39) and the PP set (*N* = 37).

Demographic data, duration and causes of FI as well as CGI scores by investigator were analysed at V-2 (Table 1). The time since first diagnosis of FI ranged from 11 to 486 months with a mean ± SD of 128.3 ± 109.96 months (10.69 ± 9.16 years) for the ITT population. In most of the patients (female and male), FI was associated with mild to severe EAS atrophy alone (43.59%), whereas in fewer patients FI was associated with EAS damage alone (41.00%) and a minority of patients suffered from FI due to both EAS damage and atrophy (15.38%). Two patients reported previous overlap repair surgery, but earlier than 6 months before screening. Notably, FI patients with EAS atrophy were significantly older (*p* < 0.05) than FI patients with EAS damage (atrophy, 74.84 ± 8.5 years; damage, 58.2 ± 14.5 years). Patient’s illness severity was judged by the investigator on the 7-point CGI assessment scale ranging from “normal, not at all ill” to “among the most extremely ill patients”. The major proportion of patients (94.9%) was assigned to the three most serious categories (“severely ill”, “markedly ill” and “amongst the most extremely ill”). Neither the complete physical examination nor ultrasound examinations revealed any pathological findings that required an exclusion of a patient from the trial.

**Table 1:** Patient demographics and baseline characteristics (V-2), *N* = 39 (ITT)

Characteristic	Value
Age (years), mean (SD)	60.6 (13.81)
Height (cm), mean (SD)	166.3 (6.82)
Weight (kg), mean (SD)	68.5 (14.53)
BMI (kg/m^2^), mean (SD)	24.7 (4.21)
Gender, *n* (%)	
Female	34 (87.2)
Male	5 (12.8)
History of anorectal surgeries (including anal sphincter surgery)^a^, *n* (%)	2 (5.13)
FI episodes	
Time since first diagnosis (months), mean (SD)	128.3 (109.96)
Cause of FI^b,c^, *n* (%)	
Muscle damage only	16 (41.03)
Atrophy only	17 (43.59)
Both	6 (15.38)
CGI score by investigator^d^	
Moderately ill	2 (5.1)
Markedly ill	14 (35.9)
Severely ill	17 (43.6)
Among the most extremely ill	6 (15.4)

*V-2* 3 months pre implantation, *N* number of values, *ITT* intention to treat, *SD* standard deviation, *BMI* body mass index, *n* number of patients, *FI* faecal incontinence, *CGI* Clinical Global Impression

^a^Earlier than 6 months before screening and no more than two surgeries (otherwise not included)

^b^Multiple answers possible

^c^According to medical history, in none of the patients was FI associated with constipation, diarrhoea, pelvic floor dysfunction, nerve damage or loss of storage capacity

^d^CGI scores: normal, not at all ill, borderline mentally ill, mildly ill, moderately ill, markedly ill, severely ill, among the most extremely ill patients [38]

### Symptoms of FI

**Fig. 2:**
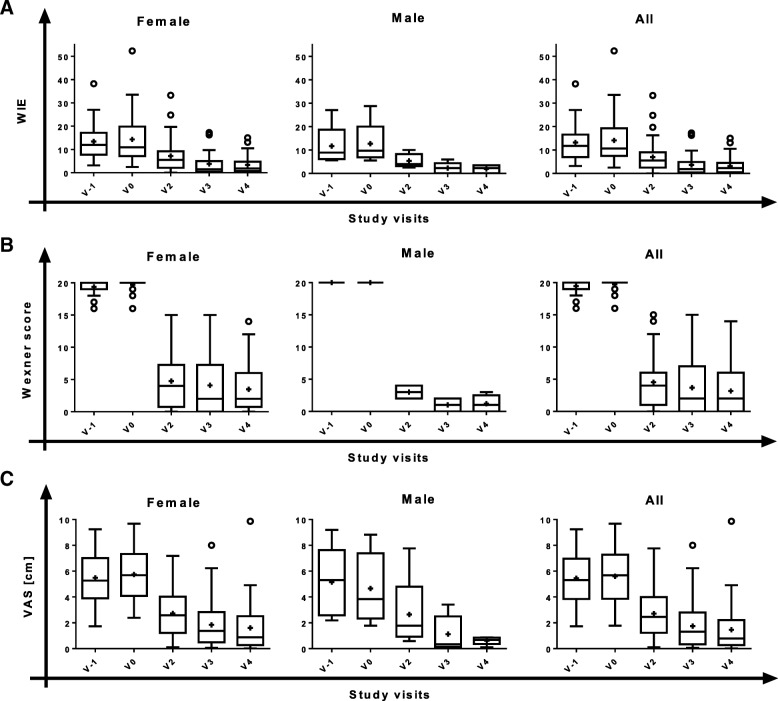
Symptoms of faecal incontinence and patient satisfaction (VAS) over study course by gender (ITT). Box plots (Tukey) for time course of (**a**) weekly incontinence episodes (WIE), (**b**) Wexner scores and (**c**) visual analogue scale (VAS) assessment. Female, *N* = 34; male, *N* = 5; all, *N* = 39. V visit, + mean, o outliers

**Fig. 3:**
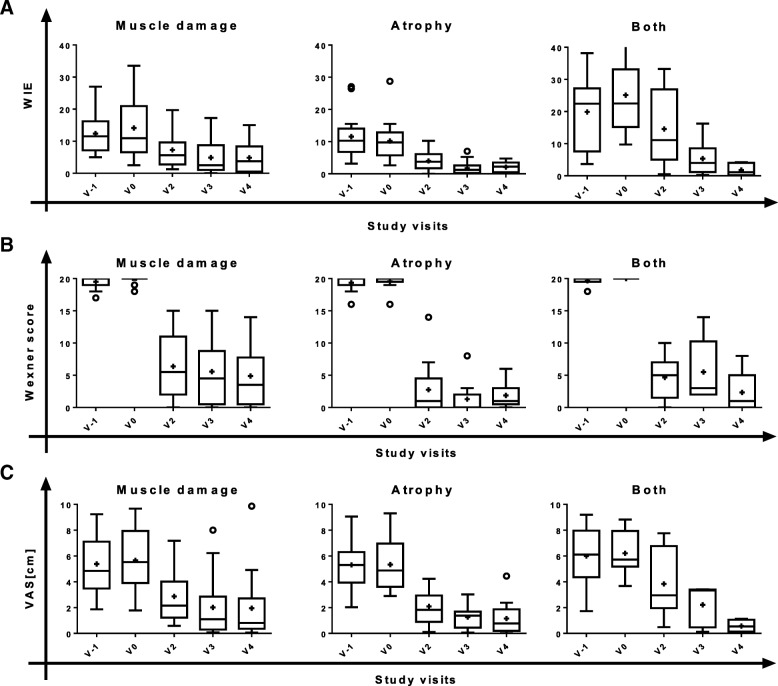
Symptoms of faecal incontinence and patient satisfaction over study course by FI cause (ITT). Box plots (Tukey) for time course of (**a**) weekly incontinence episodes (WIE), (**b**) Wexner scores and (**c**) visual analogue scale (VAS) assessment. Muscle damage, *N* = 16; atrophy, *N* = 17; all, *N* = 6. V visit, + mean, o outliers

The primary variable of the trial was the change of WIE from baseline (V0, SMDC implantation) to V2, V3 and V4. At all post-implantation visits the number of WIE was substantially reduced (Fig. 2a), indicating increasing improvement in patient’s FI status over the 12-month post-treatment period. The pre–post differences in WIE reached statistical significance in the overall ITT population and in females but not in males, possibly due to the limited number of male patients (*n* = 5) in this trial (Table 2). Furthermore, WIE was stably decreasing over time after SMDC implantation; the decrease was statistically significant independent of the cause of FI (Fig. 3a, Table 3).

**Table 2:** Pre–post differences in number of WIE, Wexner score and VAS by gender (ITT)

	Females (*N* = 34)	Males (*N* = 5)	All (*N* = 39)
WIE			
V2 – V0	−9.2 (8.20) (− 12.08; – 6.35)*	− 8.5 (7.21) (− 17.45;0.45)	−9.1 (7.99) (− 11.72; – 6.53)*
V3 – V0	− 10.6 (10.50) (− 14.32; – 6.88)*	− 10.5 (10.68) (− 23.71;2.81)	−10.6 (10.37) (− 13.99; – 7.17)*
V4 – V0	−11.0 (10.62) (− 14.66; – 7.25)*	− 10.8 (8.83) (− 21.75;0.18)	−10.9 (10.31) (− 14.27; – 7.59)*
Wexner score			
V2 – V0	−14.9 (4.85) (− 16.6; – 13.2)*	− 17.0 (1.00) (− 18.2; – 15.8)*	−15.2 (4.58) (− 16.7; – 13.7)*
V3 – V0	− 15.6 (4.56) (− 17.2; – 14.0)*	−19.0 (1.00) (− 20.2; – 17.8)*	−16.1 (4.41) (− 17.5; – 14.6)*
V4 – V0	− 16.2 (3.66) (− 17.5; – 15.0)*	− 18.8 (1.30) (− 20.4; – 17.2)*	−16.6 (3.55) (− 17.7; – 15.4)*
VAS			
V2 – V0	−4.17 (2.27) (− 5.02; – 3.32)*	− 2.59 (1.71) (− 4.71; – 0.47)*	−3.95 (2.25) (− 4.72; – 3.17)*
V3 – V0	−4.01 (2.42) (− 4.92; – 3.11)*	−3.53 (2.11) (− 6.16; – 0.91)*	−3.95 (2.35) (− 4.75; – 3.14)*
V4 – V0	−4.31 (2.65) (− 5.32; – 3.30)*	−4.04 (2.72) (− 7.42; – 0.66)*	−4.27 (2.62) (− 5.19; – 3.36)*

Data presented as mean (standard deviation) (95% confidence interval)

*WIE* weekly incontinence episodes, *VAS* visual analogue scale, *ITT* intention to treat, *N* number of values, *V* visit, *V0* implantation, *V2*, *V3* and *V4* 1, 6 and 12 months post implantation

*Significant change from baseline according to 95% confidence interval

**Table 3:** Pre–post differences in number of WIE, Wexner score and VAS by cause of FI (ITT)

	Muscle damage (*N* = 16)	Atrophy (*N* = 17)	Both (*N* = 6)
WIE			
V2 – V0	−6.83 (7.13) (− 10.6; – 3.0)*	−6.17 (5.30) (− 8.9; – 3.4)*	−10.58 (8.54) (− 19.5; – 1.6)*
V3 – V0	−9.25 (9.67) (− 14.4; – 4.1)*	−8.55 (7.12) (− 12.2; – 4.9)*	−19.79 (15.23) (− 35.8; – 3.8)*
V4 – V0	− 9.29 (10.08) (−14.7; – 3.9)*	−8.09 (5.83) (− 11.1; – 5.1)*	−23.33 (13.14) (− 19.5; – 1.6)*
Wexner score			
V2 – V0	−13.44 (5.23) (− 16.2; – 10.7)*	−16.82 (3.81) (− 18.8; – 14.9)*	− 15.33 (3.50) (− 19.0; – 11.7)*
V3 – V0	−14.25 (4.99) (− 16.2; – 10.7)*	−18.29 (2.39) (− 19.5; – 17.1)*	−14.50 (4.97) (− 19.7; – 9.3)*
V4 – V0	− 14.94 (4.42) (− 16.9; – 11.6)*	−17.71 (1.99) (− 18.7; – 16.7)*	−17.67 (3.14) (− 21.0; – 14.4)*
VAS			
V2 – V0	−2.79 (2.11) (− 3.9; – 1.7)*	−3.07 (2.40) (− 4.4; – 1.7)*	−2.37 (1.72) (− 4.2; – 0.6)*
V3 – V0	−3.65 (− 2.60) (− 5.0; – 2.3)*	−4.23 (2.41) (− 5.6; – 2.8)*	−4.10 (1.47) (− 5.9; – 2.3)*
V4 – V0	−3.71 (2.84) (− 5.2; – 2.2)*	− 4.45 (2.35) (− 5.8; – 3.1)*	−5.89 (2.45) (− 9.8; – 2.0)*

Data presented as mean (standard deviation) (95% confidence interval)

*WIE* weekly incontinence episodes, *VAS* visual analogue scale, *FI* faecal incontinence, *ITT* intention-to-treat, *N* number of values, *V* visit, *V0* implantation, *V2*, *V3* and *V4* 1, 6 and 12 months post implantation

*Significant change from baseline according to 95% confidence interval

Descriptive analyses of the change in the Wexner scores due to SMDC implantation demonstrated a rapid decrease from V0 to V2 (Fig. 2b and Fig. 3b), indicating a considerable improvement of the FI severity. This decrease was statistically significant and stable over time both in male and female patients, as well as in EAS damage (muscle damage), EAS atrophy (atrophy) and EAS damage and atrophy (both) subgroups, as revealed by the pre–post differences between baseline and all post-implantation visits (Table 2 and Table 3).

### Patients’ satisfaction and Clinical Global Impression assessment

In accordance with the improvements in FI status and FI severity, FI complaints (VAS) rapidly decreased after cell implantation in both female and male patients (Fig. 2c) and independent of FI cause (Fig. 3c). All changes from baseline to post implantation reached statistical significance (Table 2 and Table 3). Also, the patients reported severity of FI decreased significantly (all patients, mean (95% CI): V2 – V0, − 0.267 (− 0.41; – 0.12); V3 – V0, − 0.308 (− 0.48; – 0.14); V4 – V0, − 0.383 (− 0.56; – 0.20)).

The influence of FI on patients’ quality of life (QOL) was evaluated on the FIQL scale. This scale ranges from 1 to 4, with 1 indicating a lower functional status of QOL and 4 indicating not affected by FI [21]. At all post-implantation visits, higher FIQL scores compared to those before cell implantation (V-2 and V0) were observed for all four scale domains (Table 4), indicating an improved QOL. The beneficial effect appeared soon after cell implantation (at V2) with a stable trend of improvement in patients’ QOL over time. In the overall population, all pre–post differences from V0 to V2, V3 and V4 were statistically significant (Table 4). This applied also to the female population; in the male population, pre–post differences for V3 – V0 and V4 – V0 for the domain coping/behaviour reached significance (data not shown).

**Table 4:** FIQL domain scores by visit and pre–post differences (all patients, ITT)

	V-2	V0	V2	V3	V4
By visit, mean (SD)					
Lifestyle	2.1 (0.71)	2.2 (0.72)	3.2 (0.77)	3.3 (0.69)	3.4 (0.77)
Coping/behaviour	1.4 (0.34)	1.6 (0.54)	2.7 (0.77)	2.8 (0.86)	3.0 (0.91)
Depression/perception	2.3 (0.84)	2.2 (0.75)	3.2 (0.72)	3.3 (0.73)	3.3 (0.72)
Embarrassment	1.4 (0.58)	1.7 (0.66)	2.8 (0.89)	3.0 (0.80)	3.2 (0.84)
			V2 – V0	V3 – V0	V4– V0
Pre–post differences, mean (95% CI)					
Lifestyle			1.0 (0.74; 1.19)*	1.1 (0.92; 1.35)*	1.2 (0.95; 1.46)*
Coping/behaviour			1.1 (0.83; 1.35)*	1.3 (0.98; 1.55)*	1.4 (1.07; 1.67)*
Depression/perception			0.9 (0.68; 1.14)*	1.0 (0.77; 1.26)*	1.1 (0.81; 1.33)*
Embarrassment			1.0 (0.66; 1.29)*	1.2 (0.92; 1.52)*	1.4 (1.11; 1.73)*

*FIQL* Fecal Incontinence Quality of Life, *ITT* intention to treat, *V* visit, *V-2* 3 months pre implantation, *V0* implantation, *V2*, *V3* and *V4* 1, 6 and 12 months post implantation, *SD* standard deviation, *CI* confidence interval

*Significant change from baseline according to 95% CI

The rate of treatment-induced changes of patients’ FI condition was assessed by the investigator at trial end (V4) on the 7-point CGI scale ranging from “very much improved” to “very much worse” since the initiation of treatment. The results show improvement for all patients except for one for whom “no change” was reported. The vast majority of patients with improvement were assessed by the investigator as “very much improved” (29 (74.4%) patients). The assessment was “much improved” for eight (20.5%) patients and “minimally improved” for only one (2.6%) patient.

### Responders

**Fig. 4:**
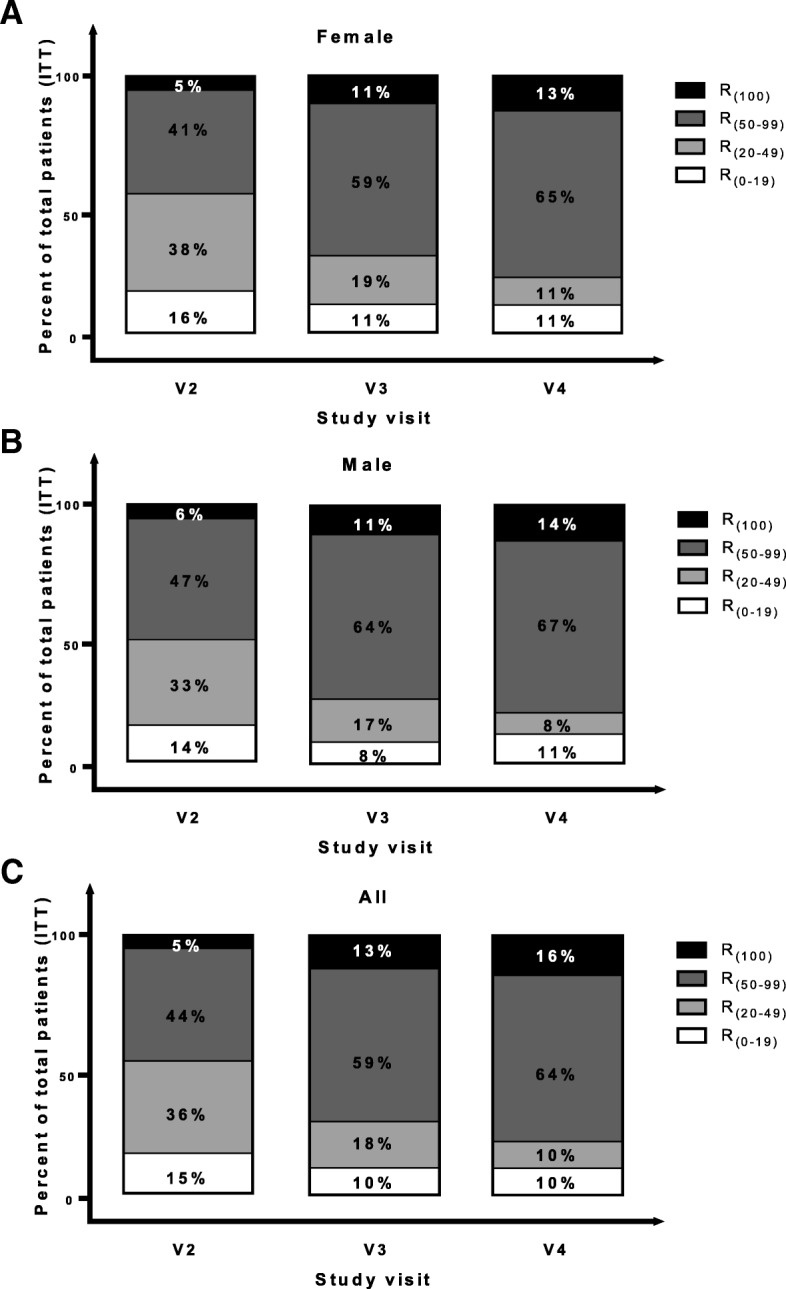
Responder rates according to decrease in weekly incontinence episodes (WIE) over study visits by gender. **a** Female (*n* = 34), **b** male (*n* = 5) and **c** all (*n* = 39) patients of the ITT set. R_(100),_ R_(50–99)_, R_(20–49)_ and R_(0–19)_, percentage of patients with decrease in WIE of 100%, 50–99%, 20–49% and 0–19%, respectively from baseline (V0) to post-implantation visits. ITT intention to treat, R response, V visit, V2, V3 and V4 1, 6 and 12 months post implantation

**Fig. 5:**
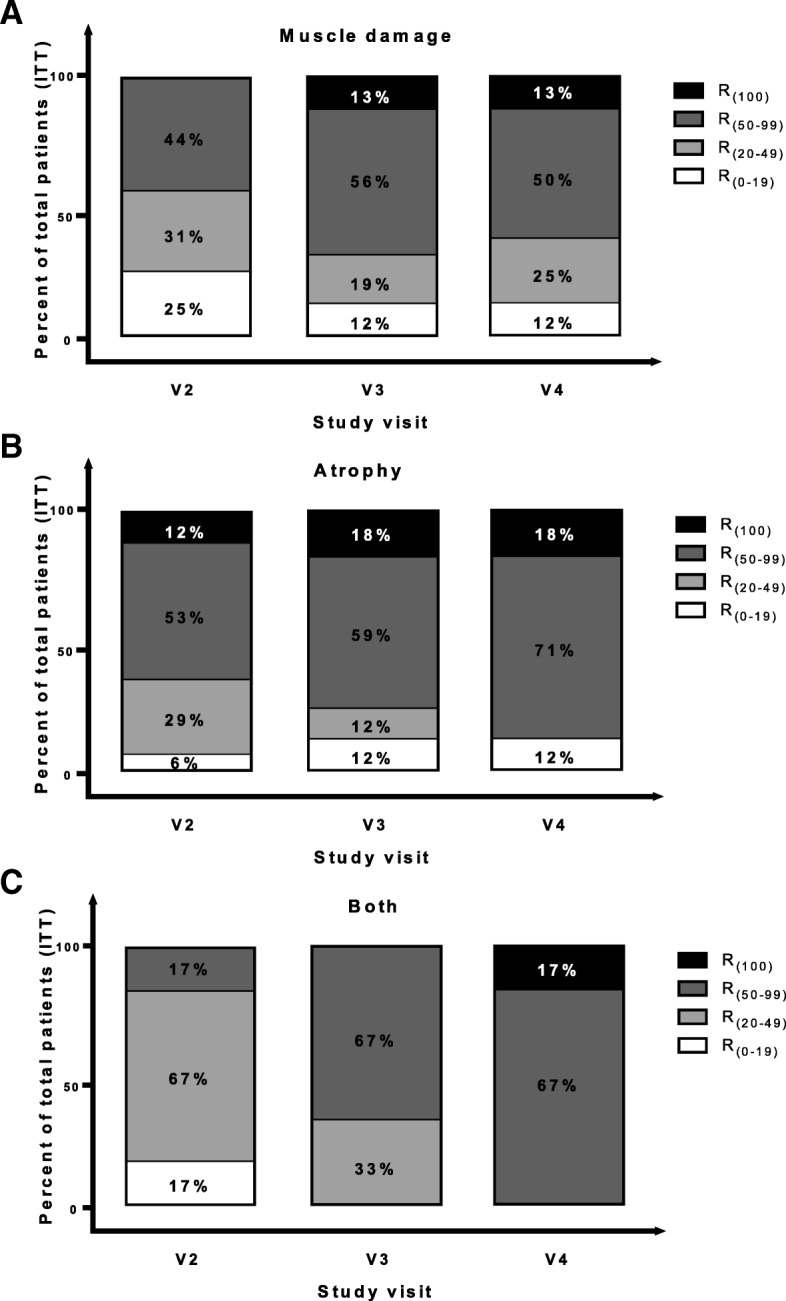
Responder rates according to decrease in weekly incontinence episodes (WIE) over study visits by cause of FI. Patients with FI due to (**a**) EAS damage (muscle damage, *N* = 16), (**b**) EAS atrophy (atrophy, *N* = 17) and (**c**) EAS damage and atrophy (both, *N* = 6). R_(100),_ R_(50–99)_, R_(20–49)_ and R_(0–19)_, percentage of patients (ITT) with decrease in WIE of 100%, 50–99%, 20–49% and 0–19%, respectively from baseline to post-implantation visits. ITT intention to treat, R response, V visit, V2, V3 and V4 1, 6 and 12 months post implantation

In all cases of defined response criteria, high responder rates were observed soon after cell implantation (at V2, 1 month post implantation) with a tendency of increasing rates until trial end (V4, 12 months post implantation) (Fig. 4). Comparing V0 and V4, in 80.6% of males and 78.4% of females as well as an overall 79.5% of patients, the WIE frequency had decreased by at least 50% (R_(50–99)_ and R_(100)_ combined) (Table 5). In 63%, 89% and 100% of patients whose FI was associated with muscle damage alone, atrophy alone and both damage and atrophy, respectively, WIE frequency decreased by at least 50% at V4 compared to V0 (Fig. 5 and Table 6). Patients who experienced complete remission of symptoms after SMDC treatment (R_(100)_) could be observed with increasing frequency at V3 and V4, indicating a trend of continuous improvement over time in the patient’s FI status (Table 5). Altogether, the responder analysis indicates a clear trend of decreasing FI severity and decreasing WIE over time in all patients.

**Table 5:** Responder rates (%) according to weekly incontinence episodes by gender (ITT)

	Female (*N* = 34)			Male (*N* = 5)			All (*N* = 39)		
	V2	V3	V4	V2	V3	V4	V2	V3	V4
R_(0–19)_	16.2	10.8	10.8	13.9	8.3	11.1	15.4	10.3	10.3
R_(20–49)_	37.8	18.9	10.8	33.3	16.7	8.3	35.9	17.9	10.3
R_(50–99)_	40.5	59.5	64.9	47.2	63.9	66.7	43.6	59.0	64.1
R_(100)_	5.4	10.8	13.5	5.6	11.1	13.9	5.1	12.8	15.4

*ITT* intention to treat, *N* number of values, *V* visit, *V2*, *V3* and *V4* 1, 6 and 12 months post implantation, *R*_*(0–19)*_, *R*_*(20–49)*_,*R*_*(50–99)*_ and *R*_*(100)*_ percentage of patients with decrease in weekly incontinence episodes of 0–19%, 20–49%, 50–99% and 100%, respectively from baseline (V0) to post-implantation visits

**Table 6:** Responder rates (%) according to weekly incontinence episodes by cause of FI (ITT)

	Muscle damage (*N* = 16)			Atrophy (*N* = 17)			Both (*N* = 6)		
	V2	V3	V4	V2	V3	V4	V2	V3	V4
R_(0–19)_	25.0	12.5	12.5	5.9	11.8	11.8	16.7	0.0	0.0
R_(20–49)_	31.3	18.8	25.0	29.4	11.8	0.0	66.7	33.3	0.0
R_(50–99)_	43.8	56.3	50.0	52.9	58.8	70.6	16.7	66.7	83.3
R_(100)_	0.0	12.5	12.5	11.8	17.6	17.6	0.0	0.0	16.7

*FI* faecal incontinence, *ITT* intention to treat, *N* number of values, *V* visit, *V2*, *V3* and *V4* 1, 6 and 12 months post implantation, *R*_*(0–19)*_, *R*_*(20–49)*_, *R*_*(50–99)*_ and *R*_*(100)*_ percentage of patients with decrease in weekly incontinence episodes of 0–19%, 20–49%, 50–99%, and 100%, respectively from baseline (V0) to post-implantation visits

### Anorectal manometry

Anorectal manometry tests at V-2 and V4 (Table 7) revealed a most prominent change in the functional length of the anal canal (high-pressure zone length), with a significant gain in length by 11.1 mm (95% CI 7.4; 14.8). Also, the increase in the threshold volumes for first sensation from V-2 to V4 reached significance. Enhanced values were observed for anal sphincter rest pressure and anal sphincter maximum squeeze pressure, too; however, these changes did not reach significance. The other parameters of the rectal sensation measures remained rather stable over time, including the rectoanal reflex (RAIR).

**Table 7:** Anorectal function parameters before and after SMDC implantation (all patients)

	Mean (SD)		Mean (95% CI)
	V-2	V4	V4 – V-2
Anal canal length (mm)	15.9 (6.37)	27.1 (9.47)	11.1 (7.4; 14.8)*
Anal sphincter rest pressure (mmHg)	66.2 (35.00)	72.5 (30.20)	7.0 (−4.63; 18.66)
Anal sphincter maximum squeeze pressure (mmHg)	121.4 (56.82)	130.7 (64.27)	8.9 (−4.09; 21.87)
Threshold volumes for first sensation (ml)	30.5 (24.49)	46.6 (22.09)	16.5 (5.1; 27.9)*
Threshold volumes for desire to defecate (ml)	105.0 (61.59)	100.3 (40.69)	1.5 (−20.7; 23.6)
Threshold volumes for urgency to defecate (ml)	111.8 (65.09)	100.3 (40.69)	−3.2 (− 25.9; 19.4)
Maximum tolerable volume (ml)	165.1 (72.98)	164.5 (55.64)	2.9 (−19.5; 25.3)

*SMDC* skeletal muscle-derived cell, *SD* standard deviation, *CI* confidence interval, *V* visit, *V-2* 3 months pre implantation, *V4* 12 months post implantation

*Significant change from baseline according to 95% CI

### Safety

In the total set (*N* = 44), 27 (61.4%) patients were affected by 57 AEs. All AEs were assessed by the investigator as not related to the SMDC treatment. Abnormal laboratory values were found in most of the patients at screening and trial end visits. The values were not clinically relevant in the majority of the cases and gave no reasons for safety concerns. No major changes in vital signs and physical examinations as well as no pathological findings were found during the trial. No complications or side effects related to the cell implantation procedure occurred at V0 and V1. All patients received SMDC treatment in time as planned. No severe adverse effects (SAEs) were seen.

### Characterization of SMDC

Residual SMDC produced for the clinical trial were analysed for expression of stem cell and myogenic markers by flow cytometry and immunostaining as well as for their differentiation potential and gene expression profile.

**Fig. 6:**
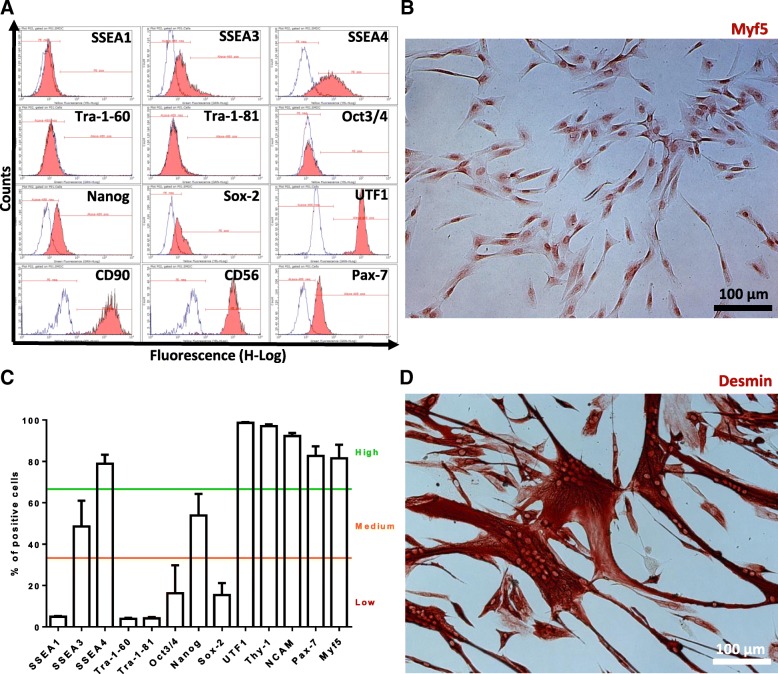
Characterization of SMDC. Histograms of flow cytometric measurements of pluripotent and muscle stem cell as well as general myogenic cell markers (**a**). Representative immunocytochemical staining of Myf5 in SMDC (**b**). Descriptive statistics of surface and intracellular markers SMDC tested for. Presented as mean ± SEM of cells derived from at least three human muscle biopsies of individual donors (**c**). Immunocytochemical desmin protein staining of SMDC in vitro differentiated to multinucleated myotubes (**d**). Scale bar = 100 μm

At least three batches of SMDC from individual human muscle biopsies were analysed for protein expression of different markers by flow cytometry and immunostaining. Mean percentages of 0–33%, 34–66% and 67–100% positive cells were categorized as low, medium and high expression for each respective marker. Expression of the pluripotent stem cell markers SSEA-1, Sox-2, Oct3/4, Tra-1-60 and Tra-1-81 [39] was found on a low percentage of SMDC (Fig. 6a, c). However, Nanog, a stem cell marker previously described to be expressed in mesenchymal stem cells [40] and functionally required for conserved myogenic differentiation potential [41], was expressed in a medium percentage of SMDC (Fig. 6). SSEA-3, SSEA-4 and CD90, all markers not only expressed on pluripotent but also mesenchymal stem cells [39, 42, 43], were found at medium (SSEA3) and high (SSEA4 and CD90) percentages (Fig. 6a, c). UTF1, a pluripotent stem cell marker correlating with *Nanog* gene expression [39], previously shown to be transcriptionally downregulated upon myotube formation of embryonic stem cell-derived myogenic cells [44], was found highly expressed in SMDC. Furthermore, analysis of the muscle stem cell markers Pax7 and Myf5 [45] as well the myogenic cell marker CD56 expressed throughout satellite cells and their descendants [46–48] revealed that a high percentage of the SMDC is positive for these markers (Fig. 6a–c).

In order to determine their differentiation potential, SMDC of all patients who received cells during the clinical trial were cultivated in differentiation medium for 4–6 days followed by immunostaining for desmin, an intermediate filament necessary for muscle formation and maintenance [49]. Every batch of cells was found to form desmin-positive multinucleated myotubes (Fig. 6d).

**Fig. 7:**
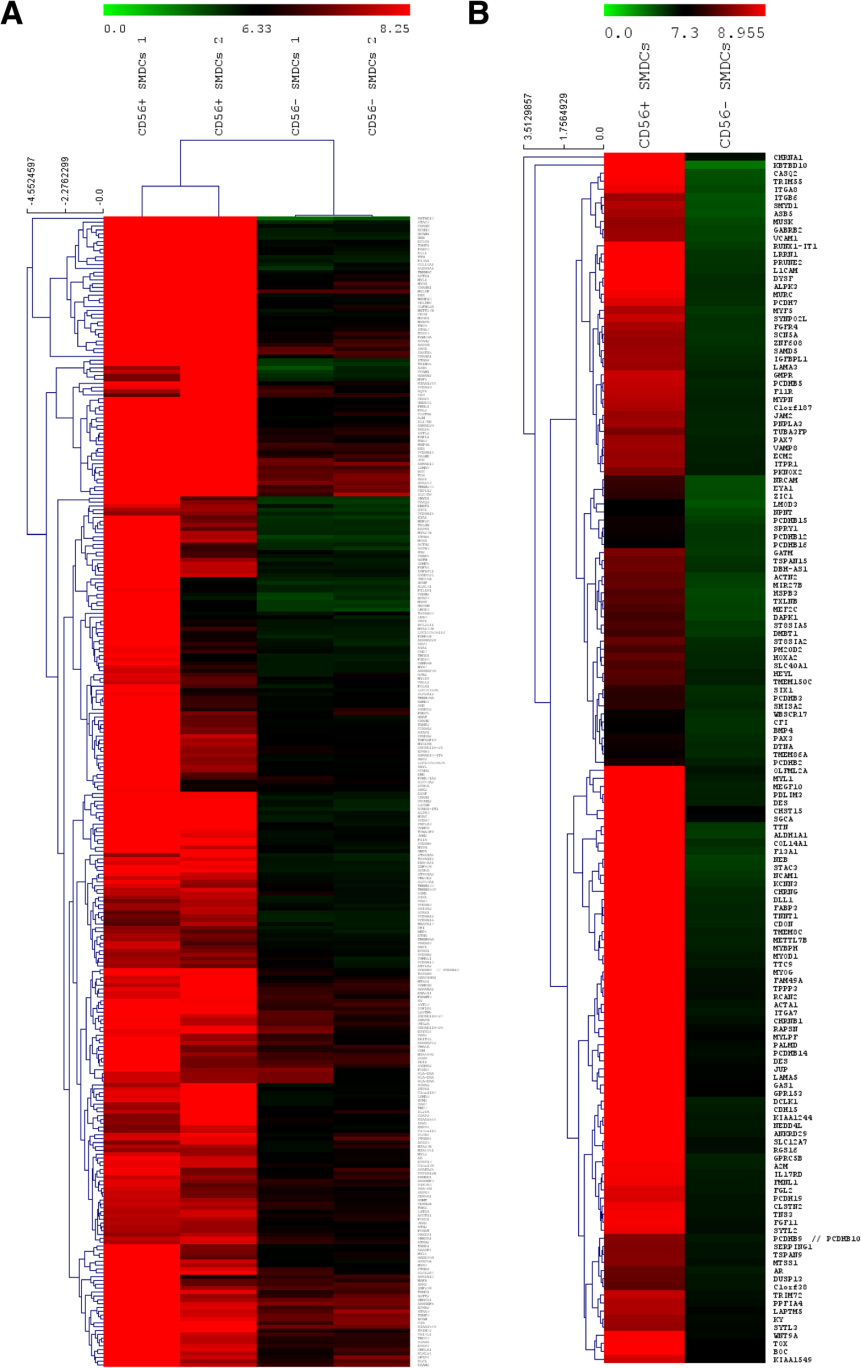
Gene expression analysis of SMDC. *k*-means cluster-identified differently expressed genes followed by Euclidean distance measure between two populations of CD56^+^ and CD56^–^ SMDC according to log_2_ signal intensities (**a**). Significance analysis of log_2_ signal intensities of differently expressed genes between CD56^+^ and CD56^–^ revealed significantly higher expressed genes in CD56^+^ than CD56^–^ cells (**b**). Log_2_ intensities of genes demonstrated as heat map followed by Euclidean distance measure. SMDC skeletal muscle-derived cells

In order to assess the myogenic commitment of the clinically used CD56^+^ SMDC on the mRNA level, gene expression was compared to CD56^–^ SMDC previously described as mesenchymal progenitors [33]. A microarray analysis was performed on RNA isolated from each two individual human muscle samples of CD56^+^ (> 95% positive) and CD56^–^ (< 5% positive) SMDC. Log_2_ intensities of all probes annotated to gene accession numbers were compared and *k*-means clustering followed by Euclidean distance measure of samples and genes in identified clusters was performed to obtain genes differentially expressed between CD56^+^ and CD56^–^ SMDC (Fig. 7a). In total, 300 differentially expressed genes were identified, of which 150 genes were found significantly higher expressed in CD56^+^ than CD56^–^ SMDC preparations (Fig. 7b). The log_2_ fold-change between-signal intensities of these genes was at least 1.43 (Table 8). Among these 150 genes significantly differently expressed between the cell types, transcripts of myogenic cell markers such as *NCAM1 (CD56), MYOD1, PAX7, PAX3, MYF5, DES (DESMIN) and MYOG* [33, 45, 47] could be identified (Table 8), thus convincingly demonstrating the myogenic commitment of CD56^+^ SMDC used for implantation into FI patients.

**Table 8:** Significantly higher expressed genes in CD56^+^ compared to CD56^–^ SMDC

Gene	CD56^+^ SMDC	CD56^–^ SMDC	Log_2_ fold change
*KBTBD10*	10.64	3.61	7.11
*CHRNA1*	12.42	6.64	5.77
*TTN*	10.81	5.37	5.63
*ALDH1A1*	10.84	5.24	5.52
*COL14A1*	10.71	5.69	5.36
*CASQ2*	9.09	4.60	5.29
*F13A1*	10.54	5.44	5.24
*NEB*	10.06	5.21	5.02
*MEGF10*	10.83	5.99	4.85
*PDLIM3*	10.95	6.13	4.85
*STAC3*	10.06	5.27	4.78
*OLFML2A*	11.19	6.53	4.77
*MYL1*	10.48	6.30	4.76
*NCAM1*	10.03	5.42	4.75
*DES*	10.92	6.13	4.75
*KCNN3*	9.95	5.56	4.57
*SMYD1*	8.52	4.66	4.51
*CHRNG*	10.18	5.84	4.46
*TRIM55*	8.94	4.68	4.42
*TMEM8C*	9.92	6.10	4.30
*LRRN1*	9.18	5.47	4.03
*DLL1*	9.63	5.84	4.01
*ACTA1*	10.01	6.46	4.00
*ITGB6*	8.37	4.54	3.99
*METTL7B*	10.01	6.02	3.99
*ITGA8*	8.86	4.87	3.99
*ASB5*	8.45	4.60	3.97
*MYOG*	10.27	6.58	3.93
*FABP3*	9.68	5.94	3.92
*PRUNE2*	9.31	5.47	3.84
*CHST15*	10.93	7.11	3.82
*TNNT1*	9.61	5.92	3.82
*SGCA*	10.66	6.93	3.75
*ITGA7*	10.14	6.38	3.75
*RUNX1IT1*	8.98	5.23	3.75
*MYBPH*	9.77	6.08	3.74
*FAM49A*	10.19	6.56	3.68
*GABRB2*	8.38	5.04	3.67
*CDON*	9.53	5.89	3.66
*NRCAM*	7.74	4.73	3.58
*TPPP3*	10.23	6.67	3.57
*RCAN2*	10.26	6.71	3.55
*MUSK*	8.33	4.86	3.54
*L1CAM*	9.04	5.49	3.54
*MYF5*	8.51	5.37	3.54
*MYOD1*	9.83	6.30	3.53
*TTC9*	9.81	6.29	3.53
*VCAM1*	8.40	4.98	3.49
*CHRNB1*	9.92	6.58	3.47
*DCLK1*	9.38	6.08	3.43
*DYSF*	8.96	5.74	3.41
*HSPB3*	7.91	4.89	3.39
*RAPSN*	10.17	6.86	3.36
*MURC*	8.90	5.62	3.31
*GPRC5B*	8.99	6.07	3.29
*SYNPO2L*	8.65	5.54	3.28
*KIAA1244*	9.30	6.37	3.26
*ALPK3*	8.99	5.75	3.25
*ACTN2*	8.02	5.20	3.20
*PCDH7*	8.78	5.60	3.19
*MYLPF*	10.04	7.05	3.19
*LMOD3*	7.26	4.50	3.15
*CDH15*	9.30	6.20	3.09
*EYA1*	7.72	4.73	3.08
*FGFR4*	8.45	5.57	3.03
*TXLNB*	7.97	5.03	3.01
*PCDHB14*	9.59	6.62	2.99
*NEDD4L*	9.32	6.34	2.99
*MIR27B*	8.05	5.10	2.98
*GATM*	8.19	5.35	2.98
*PALMD*	9.78	6.82	2.97
*MEF2C*	7.89	5.04	2.96
*DAPK1*	7.81	5.00	2.96
*A2M*	8.91	6.08	2.91
*ZIC1*	7.66	4.76	2.89
*SCN5A*	8.40	5.51	2.89
*ANKRD29*	9.15	6.40	2.82
*ZNF608*	8.34	5.54	2.81
*SAMD5*	8.39	5.67	2.81
*SLC12A7*	9.24	6.70	2.78
*PCDHB5*	8.62	5.86	2.78
*IGFBPL1*	8.41	5.77	2.77
*NPNT*	7.33	4.86	2.75
*RGS16*	9.28	6.63	2.72
*DES*	9.48	6.79	2.70
*GMPR*	8.65	6.02	2.70
*IL17RD*	8.91	6.25	2.69
*F11R*	8.57	5.92	2.65
*MYPN*	8.53	5.99	2.64
*FMNL1*	8.89	6.35	2.57
*ST8SIA5*	7.90	5.37	2.57
*LAMA3*	8.37	5.83	2.57
*C1orf187*	8.49	5.97	2.57
*TSPAN15*	8.08	5.50	2.56
*PAX7*	8.37	6.03	2.51
*DBHAS1*	8.10	5.60	2.50
*FGL2*	8.77	6.32	2.50
*DMBT1*	7.80	5.28	2.48
*JUP*	9.49	7.01	2.46
*JAM2*	8.36	5.93	2.45
*PNPLA3*	8.42	5.94	2.45
*TNS3*	8.92	6.53	2.39
*CLSTN2*	8.88	6.48	2.38
*PCDH19*	8.75	6.49	2.37
*FGF11*	8.92	6.59	2.36
*VAMP8*	8.40	6.07	2.33
*ST8SIA2*	8.05	5.75	2.31
*PCDHB15*	7.46	5.16	2.30
*TUBA3FP*	8.18	5.88	2.30
*LAMA5*	9.24	7.01	2.27
*SYTL2*	8.87	6.64	2.24
*ECM2*	8.33	6.08	2.24
*ITPR1*	8.37	6.17	2.24
*GAS1*	9.46	7.28	2.18
*HOXA2*	8.02	5.88	2.18
*PM20D2*	7.87	5.76	2.11
*SPRY1*	7.45	5.42	2.07
*PKNOX2*	8.21	6.17	2.07
*WNT9A*	8.93	6.87	2.07
*TRIM72*	8.61	6.60	2.05
*SIX1*	7.69	5.64	2.05
*SLC40A1*	7.89	5.89	2.03
*PCDHB12*	7.42	5.40	2.02
*GPR153*	9.30	7.31	2.02
*PCDHB16*	7.33	5.37	1.96
*PCDHB3*	7.64	5.70	1.93
*PCDHB9//PCDHB10*	8.25	6.35	1.89
*TOX*	9.11	7.23	1.88
*CFI*	7.39	5.53	1.86
*HEYL*	7.95	6.14	1.86
*BOC*	8.98	7.15	1.84
*TMEM150C*	7.91	6.07	1.83
*WBSCR17*	7.41	5.65	1.79
*SHISA2*	7.69	5.90	1.78
*PAX3*	7.49	5.73	1.77
*PPFIA4*	8.49	6.72	1.77
*AR*	8.06	6.30	1.76
*SERPING1*	8.21	6.47	1.76
*TSPAN9*	8.23	6.49	1.73
*BMP4*	7.29	5.58	1.72
*MTSS1*	8.25	6.60	1.67
*DTNA*	7.48	5.83	1.66
*TMEM86A*	7.40	5.78	1.63
*DUSP13*	8.04	6.43	1.62
*C1orf38*	8.01	6.44	1.57
*KY*	8.38	6.83	1.56
*LAPTM5*	8.50	6.97	1.53
*SYTL3*	8.34	6.86	1.48
*PCDHB2*	7.55	6.10	1.45
*KIAA1549*	8.70	7.27	1.44

## Discussion

The main aim of the present study was to extend the testing of ultrasound-guided SMDC injection to a population frequently affected by FI: patients suffering from FI due to EAS damage and/or especially atrophy. What is additionally needed in these cases is functional regeneration of atrophic EAS (i.e. of muscles affected by degeneration). As a consequence of degeneration, FI due to EAS atrophy is often observed with advanced age [24–26] and will occur also in patients with preceding EAS damage during aging. Our results are in line with the assumption that FI patients with EAS atrophy are usually older than patients with EAS damage. Accordingly, our total study population was clearly older (age 60.6 ± 14.0 years) than the populations in the earlier trials (age 38.3–52.0 years) [17–20].

Of the patients included in the ITT set in this trial, the majority suffered from FI associated only with EAS atrophy (43.6%), whereas fewer patients suffered from FI due to EAS damage (41.0%) and just a few patients suffered from both EAS damage and atrophy (15.4%). However, all 39 patients (female and male) in our study, regardless of FI cause, experienced significant improvements in most of the parameters evaluated, including Wexner score, WIE, response to treatment and severity of incontinence during the observation period of 1 year. Thus, it is suggested that, beside EAS damage-related FI, SMDC treatment is also a good indication in EAS atrophy-related FI, where no effective surgical option exists up to now. The finding of this study that patients suffering from FI due to EAS atrophy significantly improve in FI symptoms, together with the previous finding that treatment of patients with FI due to obstetric trauma did not result in changes in the morphology of the EAS damage (scar tissue) [17], suggest that SMDC therapy treats FI by improving the remaining muscle functionality but not by replacing scar tissue.

Treatment effects observed in the current study population are similar to the results from our pilot study on 10 women with FI due to EAS damage by obstetric trauma [17]. Two further studies by Romaniszyn et al. [20] and Boyer et al. [19] report similar results after stem cell injection for therapy of FI, with significant improvements already after 6 weeks and 6 months, respectively. After 1 year, changes were highly significant, even though the changes in comparable scores were smaller in previous studies than we observed in the present study (mean Wexner score − 4.44 [20] and median CCI − 4.5 [19] vs mean Wexner score − 16.6 (present study)). This effect might result from the use of SMDC populations with different expression of CD56, a marker for myogenic cells [47, 50] that to some extent correlates with the myogenic potency of the cells [32]. In the present study we used SMDC samples with 91.45 ± 11% CD56^+^ cells, whereas some of the SMDC samples used by Romaniszyn et al. contained less than 50% CD56^+^ cells [20] and Boyer et al. used populations with 55 ± 7% CD56^+^ cells [19]. In summary, these findings suggest that patients with EAS damage and/or atrophy may be at least equally eligible for FI treatment with SMDC as patients suffering from FI due to EAS damage alone or other causes.

Previous investigations included only a single male patient [20], so it was impossible to perform any statistical analysis on the outcome of SMDC injection in men. Although the reduction in WIE in the five male patients in the current study did not reach statistical significance, their Wexner and VAS scores did significantly decrease, suggesting that SMDC therapy might also be effective in male patients.

In the current study, both physicians and patients rated the success of the therapy very positively: as assessed by the investigator, 94.9% of all patients were in the two highest categories (“very much”, “much improved”) of the 7-point CGI [38] 1 year after implantation. As assessed by the patients, both IE and QOL (FIQL) had already improved significantly in the first month after implantation, with a significant decrease in FI complaints (VAS). These improvements were sustained until the end of this study (1 year). Data from our pilot study show that improvement is maintained in all FIQL categories even 5 years post implantation [17, 18]. Such significant and long-lasting improvement in QOL is of particular importance for the patients, because FI poses a strong burden to the affected by leading to shame, depression, need to reorganize daily life, withdrawal from social activities and so forth [51].

Despite the positive outcome in terms of symptoms, we observed hardly any physiological changes (manometry, ultrasound), particularly in mean and maximum resting and squeeze pressure 1 year post implantation. This corresponds with observations by Boyer et al. [19] and with results from our pilot study [17]. In contrast, Romaniszyn et al. [20] reported significant changes in resting and squeeze pressures over time (after 1 year). This could be explained by the fact that they also observed an increase of the amplitude of the motor unit potential (MUP) on electrical stimulation by electromyography, suggesting an increase in muscle strength of the single muscle fibres with time [20]. This might also be an explanation for what we observed in our pilot study where both resting and squeeze pressures increased after 2 and 5 years [18]. Also, the increase of the anal canal length (high-pressure zone) with time as seen in the pilot study and the current study could be attributed to strengthened muscle fibres. Hence, it would be plausible that the patients in the current study and in the study by Boyer et al. [19] will have increased anal pressures in the future. Recently, we demonstrated that AChE activity of differentiating SMDC correlates with their in-vitro fusion competency and that transplantation of high AChE SMDC results in increased treatment efficacy following intrasphincteric implantation in FI patients [32]. Thus, SMDC are suggested to regenerate weakened EAS in FI patients due to formation of new myofibres and/or fusion with existing myofibres. In line with this suggestion, transplantation of reporter protein-expressing muscle-derived cells into mouse skeletal muscle resulted in reporter protein expression within host myofibres [52, 53]. Animal studies revealed that especially the transplantation of Pax7^+^ and Myf5^+^ muscle stem cells might provide long-term efficacy in skeletal muscle regeneration as these cells, besides differentiation and fusion to myofibres, are able to repopulate the satellite cell niche within host muscle [45, 54]. SMDC isolated according to the protocols of this and a previous clinical trial [18] were found not only to harbour myogenic gene expression profile, myogenic marker expression (CD56) and in-vitro fusion competency necessary for differentiation and myofibre formation, but also to express several stem cell markers found on mesenchymal stem cells (CD90, SSEA-4, SSEA-3) as well as muscle stem cells (Pax7^+^, Myf5^+^, UTF1). Thus, some of the intramuscularly injected Pax7^+^ cells might de-differentiate and regain a quiescent satellite cell phenotype as demonstrated in isolated myofibres [54], which might explain the long-lasting effects of SMDC therapy found over the study course of 1 year in this and other studies [19, 20] as well as over 5 years in our previous study [18]. However, the real fate of SMDC in the human setting of intrasphincteric implantation for FI treatment remains elusive. Moreover, additional mechanisms of SMDC therapy such as paracrine effects might also play a role and thus studies on the detailed mode of action of SMDC for skeletal muscle regeneration are highly requested.

Our data clearly demonstrate that satisfactory results can be achieved even with a lower cell count than used in previous studies (previous studies 249 ± 68 × 10^6^ cells [20], 100 ± 20 × 10^6^ cells [19] and 121 ± 12 × 10^6^ cells [17] vs current study 79.4 ± 22.5 × 10^6^ cells/patient). Furthermore, cryo-preservation of the cells appears to have no detrimental effect on the outcome when compared to data from studies using freshly prepared cells [17, 19, 20]. This also agrees with the first attempts by Boyer et al. [19], who observed a response in 6 of 10 patients treated with cryo-preserved cells. The use of cryo-preserved cells is a major advance in terms of practicability of the method and will considerably simplify their use in clinical practice in the future.

The major limitations of our study were that it was unblinded and uncontrolled, so that placebo effects cannot be ruled out. The very idea of receiving potential FI therapy might already have had a positive impact on the QOL of study participants. In fact, Boyer et al. [19] observed in their placebo group (sham injection) a transient decrease in CCI scores 6 months post implantation that disappeared again by 12 months. The authors explained this as a possible placebo effect due to an initial bulking effect of the injection by itself. It is also possible that the small injuries to the tissue by the needle stimulated endogenous regeneration in the placebo patients. Although our study included the largest study group (39 patients) of all trials to date (10–12 patients receiving SMDC [17, 19, 20]), thus strengthening the current knowledge, our study lacks a placebo control. Boyer et al.’s placebo-controlled phase II trial found significant differences in secondary endpoints between cell and placebo treatment. Since changes in physiological parameters were only observed during the long-term (5-year) follow-up of our pilot study [18], long-term results from this study should confirm our expectations.

## Conclusions

Ultrasound-guided injection of autologous SMDC into the external sphincter of patients suffering from FI due to EAS damage and/or atrophy achieved significant improvement of FI symptoms and QOL, with excellent tolerability. Also, SMDC treatment was shown for the first time to yield significant improvements of VAS and Wexner score in male patients. Convincingly, SMDC treatment of FI was effective also in patients suffering from EAS atrophy, which is a big step forward. Compared to surgical interventions, SMDC injection is minimally invasive with a relatively low burden for the patients bearing the possibility to become the first-line treatment option for patients with faecal incontinence.
